# Associations of Insomnia With Hypertension and Coronary Artery Disease Among Patients With Type 2 Diabetes Mellitus

**DOI:** 10.3389/fcvm.2021.730654

**Published:** 2021-11-19

**Authors:** Yonghui Hu, Zhiyue Yan, Zhenrui Fu, Congqing Pan

**Affiliations:** ^1^National Health Commission (NHC), Key Laboratory of Hormones and Development, Tianjin Key Laboratory of Metabolic Diseases, Chu Hsien-I Memorial Hospital & Tianjin Institute of Endocrinology, Tianjin Medical University, Tianjin, China; ^2^Department of Urology, Affiliated Zhongda Hospital of Southeast University, Southeast University, Nanjing, China

**Keywords:** type 2 diabetes mellitus, insomnia, coronary artery disease, hypertension, propensity score matching

## Abstract

**Purpose:** This study aimed to determine whether insomnia is associated with hypertension (HBP) and coronary artery disease (CAD) in a hospital-based sample of patients with type 2 diabetes mellitus (T2DM).

**Methods:** Our present study included 354 patients with T2DM. According to the diagnostic criteria of insomnia, the participants were assigned to three groups based on the duration of T2DM and insomnia diagnosis. Patients with T2DM alone were placed in group A; patients with T2DM longer than insomnia were placed in group B; and patients with insomnia longer than T2DM were placed in group C. Medical history was collected from all the patients in detail. Besides, the participants underwent thorough physical examinations and laboratory measurements. Propensity score matching (PSM) was applied to evaluate the associations of insomnia with HBP and CAD. The univariate and multivariate logistic regression analysis was used to explore whether insomnia was a risk factor for HBP and CAD in patients with T2DM.

**Results:** Of 354 patients, 225 patients were included in group A, 62 patients were included in group B, and 67 patients were included in group C. Compared with groups B and C, group A showed a lower prevalence of HBP and CAD (*p* < 0.05). In addition, compared with group B, group C showed no difference in the prevalence of HBP and CAD (*p* > 0.05). After PSM was performed, groups B and C had a higher prevalence of HBP and CAD (*p* < 0.05) than group A with no significant difference between groups B and C (*p* > 0.05). In the univariate and multivariate logistic regression analysis, insomnia was a risk factor for HBP [univariate: odds ratio (OR) = 3.376, 95% CI 2.290–6.093, *p* < 0.001; multivariate: OR = 2.832, 95% CI 1.373–5.841, *p* = 0.005] and CAD (univariate: OR = 5.019, 95% CI 3.148–8.001, *p* < 0.001; multivariate: OR = 5.289, 95% CI 2.579–10.850, *p* < 0.001).

**Conclusion:** T2DM combined with insomnia was related to HBP and CAD and insomnia was a risk factor for HBP and CAD in patients with T2DM. However, larger, prospective studies are required to confirm our findings.

## Introduction

Currently, diabetes mellitus influences more than 463 million people worldwide and is one of the most important public health issues (1). The number may well-increase on account of the rising obesity rate, which is a major cause of the increase in the global incidence of type 2 diabetes mellitus (T2DM) (2). It is well-established that T2DM deeply compromises the cardiovascular system, kidneys, nerves, and eyes and is strongly associated with a series of complications. Cardiovascular events, heart failure, and atherosclerosis are mainly caused by damage to the macrovasculature. These complications are the primary cause of morbidity and mortality risks in patients with T2DM.

Insomnia is an exceedingly prevalent illness and is defined as difficulty in going to sleep or staying asleep (3), resulting in adverse effects on health of the patients and quality of life. Over the last couple of years, numerous studies have suggested relationships between insomnia and decreased acute insulin reaction to glucose, glucose metabolism disorder, and insulin resistance, introducing it as a robust contributor to T2DM. The prevalence of insomnia in patients with T2DM has been reported over 40% and compared with the general population, the prevalence of insomnia was higher in patients with T2DM (4) As there are reasonable biological mechanisms linking insomnia to the risk of developing T2DM through increasing insulin resistance and appetite, insomnia can be properly treated as a potentially promising approach to reduce T2DM incidence (5). Besides, increased inflammation and sympathetic activation are activated because sleep disruption is related to insulin resistance and ultimately to T2DM (6). Insomnia has also been reported to accelerate the loss of β-cell function and increase β-cell apoptosis (7). Also, low subjective sleep quality and increased subjective insomnia symptoms (8) have been associated with higher glycosylated hemoglobin A1c (HbA1c) in patients with T2DM.

These publications indicate that glucose metabolism might be adversely affected by insomnia. However, few studies have investigated whether insomnia is related to hypertension (HBP) and coronary artery disease (CAD) in patients with T2DM. Therefore, this study aimed to examine whether insomnia is connected with these cardiovascular complications in a hospital-based sample of patients with T2DM.

## Methods

### Study Subjects

From September 2019 to August 2020, we recruited 406 patients from the Tianjin Medical University Chu Hsien-I Memorial Hospital; a total of 354 patients meet the inclusion criteria, including age ≥ 18 years, diagnosed with T2DM according to the WHO (1999) criteria, ie, fasting blood glucose (FBG) ≥ 7.0 mmol/L and/or 2-h postprandial blood glucose (PBG) ≥ 11.1 mmol/L. The patients were divided into three groups: patients with T2DM only were assigned to group A (the T2DM-alone group); if the duration of T2DM was longer than insomnia, the patients were assigned to group B (T2DM combined with insomnia group); and if the duration of insomnia was longer than T2DM, the patients were assigned to group C (the insomnia combined with T2DM group).

### Determination of Main Indicators

The presence of insomnia was evaluated according to the World Mental Health Survey Initiative Version of the WHO Composite International Diagnostic Interview (WMH-CIDI) (9). It is well-established that the Composite International Diagnostic Interview (CIDI) is a standardized approach designed to be used in epidemiological research and can provide the diagnosis based on the fourth edition of the Diagnostic and Statistical Manual of Mental Disorders (DSM-IV) and the International Classification of Diseases, Tenth Revision (ICD-10) criteria. The following issues, included in the WMH-CIDI, were asked from all the patients: “In the past 12 months, have you ever experienced, in a period of 2 weeks or longer, any of the following situations: (1) Problems in initiating sleep, taking you 2 h or more to fall asleep almost every night; (2) Problems in staying asleep when you woke up almost every night and took an hour or longer to go back to sleep; (3) Problems in waking up too early; did you wake up at least 2 h earlier than you planned to almost every morning?” If at least one of these questions was answered positively, the patients were classified as having insomnia or if all the answers were negative, the patients were classified as having no insomnia. In addition, another question from the WMH-CIDI (“In the past 12 months, have you ever experienced troubles feeling sleepy during the day for 2 weeks or longer”) was used to screen for other potential undiagnosed sleep-arousal diseases (such as obstructive sleep apnea) in the included patients. Individuals without insomnia, who responded positively to this question, were excluded from this study.

Patients were diagnosed as with CAD if stenosis was detected in ≥50% of the lumen diameter of a primary coronary artery, according to cardiac CT examination (10). Moreover, HBP was diagnosed as systolic blood pressure (SBP) ≥140 mm Hg and/or diastolic blood pressure (DBP) ≥90 mm Hg measured by a mercury sphygmomanometer, at rest in a sitting position in at least three separate casual measurements, in the light of the European Society of Hypertension/European Society of Cardiology 2007 guidelines (11).

### Body Measurements and Laboratory Examinations

Height and weight were measured by previously standardized methods (12). Body mass index (BMI) was defined as weight (kg)/height^2^ (m^2^). Blood samples were obtained after overnight fasting. The following laboratory indices were measured: total triglycerides (TGs), total cholesterol (TC), high-density lipoprotein (HDL), low-density lipoprotein (LDL), very low-density lipoprotein (VLDL), serum creatinine (Scr), blood urea nitrogen (BUN), HbA1c, and FBG.

### Statistical Analysis

As for continuous variables, the assumption of normality was tested for each result of interest. When data coincided with normal distribution, the independent *t*-test was conducted to compare the variables between each of the two comparisons (group A vs. group B, group A vs. group C, and group B vs. group C) and the results were reported as mean ± SD. When data did not comply with the normal distribution, the variables were compared with the Mann–Whitney *U*-test and presented as median [interquartile range (IQR)]. In addition, as for categorical variables, we selected the chi-squared test to compare the differences between each of the two comparisons in demographic and clinical variables and the results were reported as percentages. Finally, for the primary outcome (the rate of HBP and CAD), the mean differences (two-sided 95% CI) for the two comparisons were reported.

As this was a retrospective study that might be affected by the selection bias, propensity score matching (PSM) was applied to evaluate the associations of insomnia with HBP and CAD. We conducted an additional analysis through a 1:1 PSM with age, sex, BMI, smoking, and alcohol use history. We calculated odds ratios (OR) and 95% CIs to assess the association of insomnia with HBP and CAD by using the univariate and multivariate logistic regression analysis in three models [model 1: adjusted age, gender, BMI, smoking, and alcohol use; model 2: adjusted model 1+ (family history of T2DM, TG, TC, HDL, LDL, VLDL, BUN, Scr, HbA1c, FBG, and insulin therapy); and model 3: model 2 + (duration of T2DM > duration of insomnia)].

We carried out all the analyses by the IBM SPSS version 23.0, Chicago IL, USA, for windows. *p* < 0.05 was regarded as statistically significant.

## Results

### Clinical and Laboratory Characteristics of Participants

Overall, 225 patients were divided into group A (T2DM-alone group), 62 patients were assigned to group B (T2DM combined with insomnia), and 67 patients were assigned to group C (insomnia combined with T2DM). Furthermore, 129 patients with T2DM had insomnia. Table 1 presents the baseline clinical and laboratory characteristics of the patients in groups A, B, and C. The duration of T2DM was 8.00 (12.00) years, 15.00 (10.00) years, and 5.00 (6.00) years in the three groups, respectively. The duration of insomnia was 7.00 (6.00) years in group B and 10.00 (7.00) years in group C.

**Table 1:** Clinical and laboratory characteristics of study participants before propensity score matching.

**Variables**	**Group A**	**Group B**	**Group C**
	**(*n* = 225)**	**(*n* = 62)**	**(*n* = 67)**
Age(years)	55.16 ± 11.87	58.58 ± 9.05^#^	55.99 ± 10.12
Gender Male (%)	137 (60.9)	14 (22.6) ^#^	30 (44.8)^#,*^
Family history of T2DM (%)	154 (68.4)	48 (77.4)	49 (73.1)
BMI (kg/m^2^)	26.30 ± 3.57	26.86 ± 3.55	27.34 ± 4.99
SBP (mmHg)	133.76 ± 18.28	143.44 ± 21.70^#^	138.18 ± 20.02
DBP (mmHg)	80.61 ± 9.81	82.19 ± 11.25	84.43 ± 10.47^#^
TG (mmol/L)	1.81 (1.78)	1.66 (1.38)	1.77 (2.09)
TC (mmol/L)	4.98 (1.43)	4.98 (1.69)	4.80 (1.60)
HDL (mmol/L)	1.19 (0.45)	1.12(0.24)	1.19 (0.45)
LDL (mmol/L)	3.23 (1.09)	3.21 (1.01)	2.93 (1.42)
VLDL (mmol/L)	0.60 (0.43)	0.64 (0.34)	0.64 (0.59)
BUN (mmol/L)	5.43 (1.87)	5.95 (2.44)^#^	5.42 (2.63)
Scr (umol/L)	63.00 (22.50)	71.20 (20.12)^#^	66.40 (20.80)^*^
HbA1c (%)	8.30 (3.25)	8.80 (2.10)	9.10 (3.10)^#^
Duration of T2DM (years)	8.00 (12.00)	15.00 (10.00)^#^	5.00 (6.00)^#,*^
Duration of Insomnia (years)	–	7.00 (6.00)	10.00 (7.00)^*^
Smoking (%)	115(51.1)	30(48.4)	32 (47.8)
Alcohol use (%)	84(37.3)	30(48.4)	20 (29.9)^*^
FBG (mmol/L)	11.04 ± 2.93	10.78 ± 3.37	11.53 ± 4.51
Insulin therapy (%)	142(63.1)	40(64.5)	43(64.2)

# *p < 0.05 vs. group A*.

* *p < 0.05 vs. group B*.

*SBP, systolic blood pressure; DBP, diastolic blood pressure; BMI, body mass index; TG, triglyceride; TC, total cholesterol; HDL, high-density lipoprotein cholesterol; LDL, low-density lipoprotein cholesterol; VLDL, very low-density lipoprotein cholesterol; BUN, blood urea nitrogen; Scr, serum creatinine; HbA1c, glycosylated hemoglobin A1c; FBG, fasting blood glucose; HBP, hypertension; CAD, coronary artery disease*.

Compared with group A, group B patients were older with a higher rate of females and higher levels of BUN and Scr and group C had a higher rate of females and a higher level of HbA1c. Compared with group B, group C had a higher rate of males, alcohol use history, and lower level of Scr (Table 1; *p* < 0.05). There were no significant differences in the family history of T2DM, BMI, TG, TC, HDL, LDL, and VLDL between the three groups (Table 1; *p* > 0.05).

After PSM, all the baseline characteristics in patients with T2DM were balanced in every two groups (*p* > 0.05) except for the duration of T2DM and insomnia (*p* < 0.05). The level of Scr remained unbalanced between group B and group C (*p* < 0.05), which was added as a covariate in the multivariate logistic regression analysis (Table 2).

**Table 2:** Clinical and laboratory characteristics of study participants after propensity score matching.

**Variables**	**PSM 1**		**PSM 2**		**PSM 3**	
	**Group A (*n* = 55)**	**Group B (*n* =55)**	**Group A (*n* = 64)**	**Group C (*n* =64)**	**Group B (*n* = 49)**	**Group C (*n* =49)**
Age (years)	58.84 ± 12.21	57.96 ± 9.13	55.34 ± 13.55	55.91 ± 10.32	56.98 ± 8.78	56.20 ± 9.91
Gender male (%)	21 (38.2)	14 (25.5)	39 (60.9)	30 (53.1)	12 (24.5)	12 (24.5)
Family history of T2DM (%)	41 (74.5)	42 (76.4)	48 (75.0)	46 (71.9)	36 (73.5)	34 (69.4)
BMI (kg/m^2^)	26.54 ± 3.73	26.68 ± 3.28	27.80 ± 3.66	26.80 ± 4.25	26.82 ± 3.45	27.08 ± 5.23
SBP (mmHg)	136.87 ± 17.85	142.05 ± 19.92	137.50 ± 19.12	137.31 ± 19.96	142.27 ± 21.39	139.51 ± 19.10
DBP (mmHg)	81.31 ± 8.69	82.42 ± 11.73	82.30 ± 10.29	84.17 ± 10.65	83.08 ± 11.82	85.06 ± 10.31
TG (mmol/L)	2.18 (2.29)	1.56 (1.45)	2.10 (2.62)	1.78 (2.06)	1.73 (1.22)	1.78 (2.22)
TC (mmol/L)	5.19 (1.16)	4.88 (1.65)	5.07 (1.25)	4.88 (1.64)	4.82 (1.81)	4.70 (1.54)
HDL (mmol/L)	1.22 (0.45)	1.11(0.23)	1.10 (0.38)	1.21 (0.34)	1.10 (0.27)	1.10 (0.37)
LDL (mmol/L)	3.31 (1.09)	3.14 (1.07)	3.21 (0.87)	2.99 (1.51)	3.14 (1.21)	2.93 (1.31)
VLDL (mmol/L)	0.68 (0.52)	0.63 (0.28)	0.69 (0.54)	0.64 (0.59)	0.64 (0.33)	0.65 (0.54)
BUN (mmol/L)	5.54 (1.88)	5.85 (2.31)	5.45 (2.07)	5.44 (2.68)	5.85 (2.37)	5.44 (2.63)
Scr (umol/L)	60.40 (33.60)	69.40 (19.80)	67.70 (27.90)	66.40 (20.53)	71.50 (20.30)	63.80 (20.60) ^*^
HbA1c (%)	8.08 ± 1.916	8.67 ± 1.62	8.64 ± 2.48	9.51 ± 3.18	8.61 ± 1.65	9.15 ± 2.44
Duration of T2DM (years)	10.00 (12.00)	15.00 (10.00)^#^	10.00 (11.80)	5.00 (6.00)^#^	14.00 (7.50)	5.00 (6.00)^*^
Duration of insomnia (years)	–	7.00 (6.00)	–	7.00 (6.00)	7.00 (6.00)	10.00 (6.00)^*^
Smoking (%)	23 (41.8)	25 (45.5)	27 (42.2)	32 (50.0)	23 (46.9)	24 (49.0)
Alcohol use (%)	30 (54.5)	23 (41.8)	22 (34.4)	19 (29.7)	19 (38.8)	20 (40.8)
FBG (mmol/L)	10.69 ± 3.13	10.83 ± 3.56	11.18 ± 2.87	11.48 ± 4.54	10.45 ± 3.28	11.49 ± 4.87
Insulin therapy (%)	26(47.3)	35(63.6)	34 (53.1)	41 (64.1)	30 (61.2)	29 (59.2)

# *p < 0.05 vs. group A*.

* *p < 0.05 vs. group B*.

*PSM 1, propensity score matching between group A and group B; PSM 2, propensity score matching between group A and group C; PSM 3, propensity score matching between group B and group C; SBP, systolic blood pressure; DBP, diastolic blood pressure; BMI, body mass index; TG, triglyceride; TC, total cholesterol; HDL, high-density lipoprotein cholesterol; LDL, low-density lipoprotein cholesterol; VLDL, very low-density lipoprotein cholesterol; BUN, blood urea nitrogen; Scr, serum creatinine; HbA1c, glycosylated hemoglobin A1c; FBG, fasting blood glucose; HBP, hypertension; CAD, coronary artery disease*.

### Prevalence of HBP and CAD in the Three Groups

**Figure 1:**
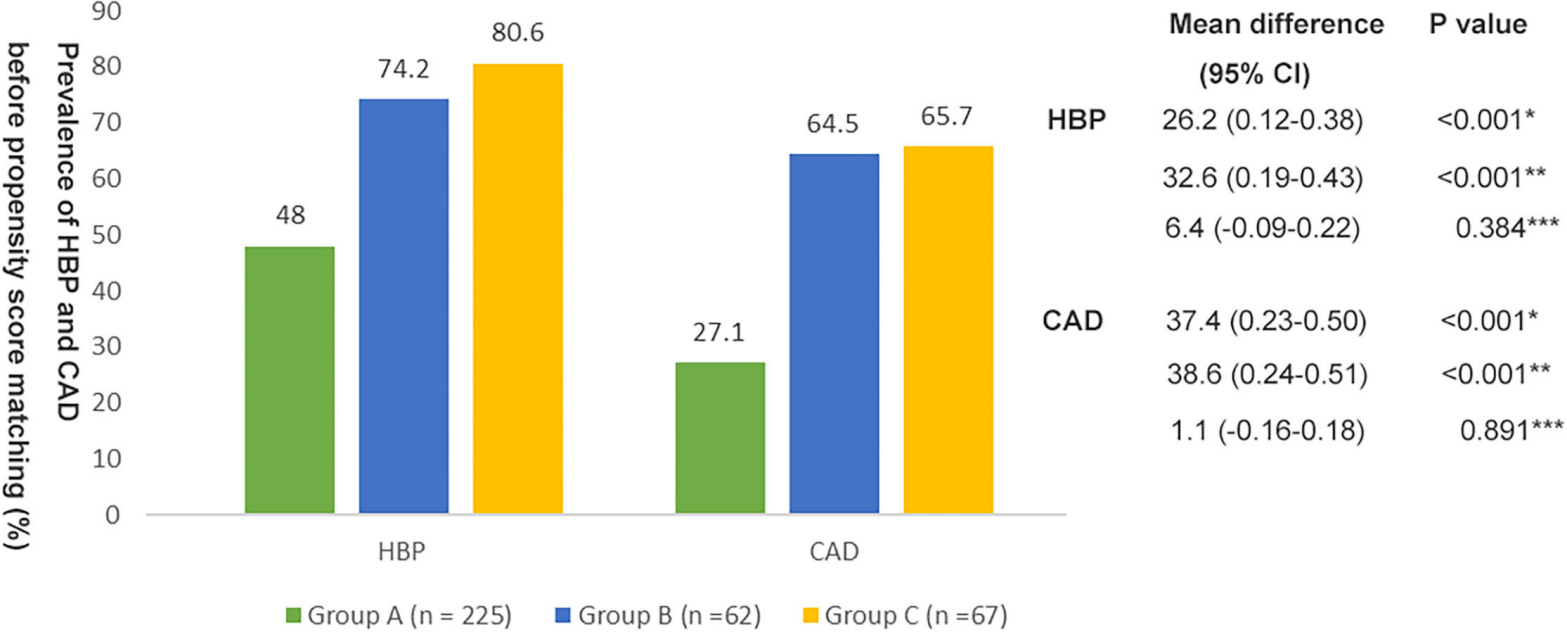
Prevalence of hypertension (HBP) and coronary artery disease (CAD) in the three groups before propensity score matching. *Group A vs. Group B; **Group A vs. Group C; ***Group B vs. Group C.

As shown in Figure 1, 48% of patients (*n* = 108) in group A, 74.2% of patients (*n* = 46) in group B, and 80.6% of patients (*n* = 54) in group C had HBP. The mean difference between group A and group B was−26.2% (95% CI, 0.12–0.38; *p* < 0.001) and the mean difference between group A and group C was−32.6% (95% CI, 0.19–0.43; *p* < 0.001). However, there was no significant difference in the prevalence of HBP between groups B and C (*p* > 0.05). 27.1% of patients (*n* = 61) in group A, 64.5% of patients (*n* = 40) in group B, and 65.7% of patients (*n* = 44) in group C had CAD. The mean difference between group A and group B was −37.4% (95% CI, 0.23–0.50; *p* < 0.001) and the mean difference between group A and group C was −38.6% (95% CI, 0.24–0.51; *p* < 0.001). However, there was no significant difference in the prevalence of CAD between groups B and C (*p* > 0.05).

**Figure 2:**
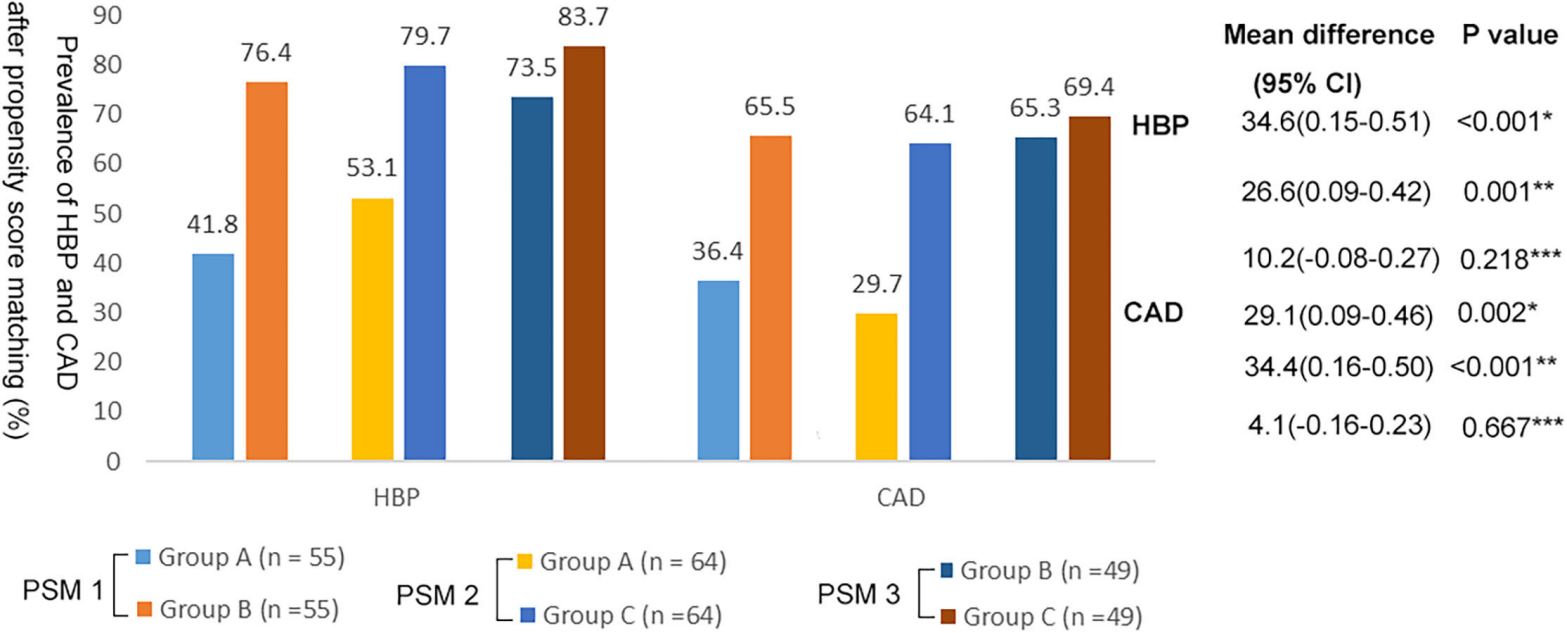
Prevalence of HBP and CAD in the three groups after propensity score matching. *Group A vs. Group B; **Group A vs. Group C; ***Group B vs. Group C.

These differences remained significant after PSM. Compared with group A, groups B and C had a higher prevalence of HBP and CAD (Figure 2; *p* < 0.05). There was no significant difference in the prevalence of HBP and CAD between groups B and C (Figure 2; *p* > 0.05).

### Causal Relationship of Insomnia With HBP and CAD in Patients With T2DM

**Figure 3:**
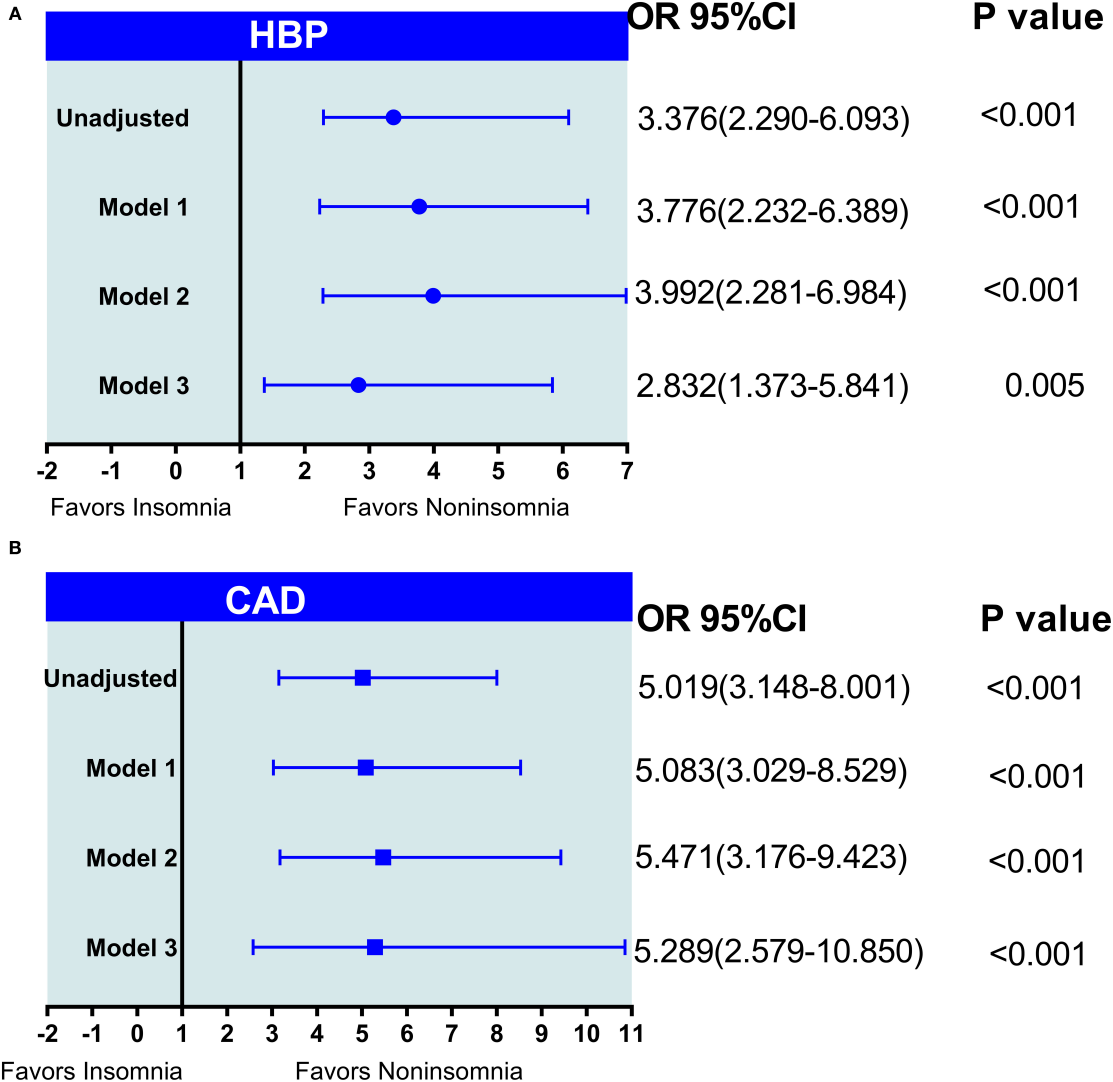
**(A,B)** The logistic regression analysis of insomnia with HBP and CAD in patients with type 2 diabetes mellitus (T2DM). Model 1: adjusted age, gender, body mass index (BMI), smoking, and alcohol use; model 2: adjusted model 1+ (family history of T2DM, TG, triglyceride; TC, total cholesterol; HDL, high-density lipoprotein; LDL, low-density lipoprotein; VLDL, very low-density lipoprotein; BUN, blood urea nitrogen; Scr, serum creatinine; HbA1c, glycosylated hemoglobin A1 c; FBG, fasting blood glucose, and insulin therapy); model 3: model 2 + (duration of T2DM > duration of insomnia).

As shown in Figure 3, in the univariate logistic regression analysis, insomnia was associated with a higher risk of HBP (OR = 3.376; 95% CI, 2.290–6.093; *p* < 0.001) (Figure 3A) and CAD (OR = 5.019; 95% CI, 3.148–8.001; *p* < 0.001) (Figure 3B). After adjustment for the natural characteristics of the body and the characteristics of laboratory indices, the ratios of HBP prevalence (model 1: OR = 3.776; 95% CI, 2.232–6.389, *p* < 0.001; model 2: OR = 3.992; 95% CI, 2.281–6.984; *p* < 0.001) and CAD (model 1: OR = 5.083; 95% CI, 3.029–8.529, *p* < 0.001; model 2: OR = 5.471; 95% CI, 3.176–9.423; *p* < 0.001) was increased. In the additional model (model 3) that added duration of T2DM longer than insomnia to the adjusted model, the ratios of HBP prevalence (OR = 2.832; 95% CI, 1.373–5.841; *p* = 0.005) and CAD (OR = 5.289; 95% CI, 2.579–10.850; *p* < 0.001) decreased slightly, but were still statistically significant.

## Discussion

This study is a hospital-based retrospective study to analyze the associations of insomnia with HBP and CAD in adult patients with T2DM in northern China by using an acknowledged insomnia scaling system. Our findings suggested that group A (T2DM-alone group) had a lower prevalence of HBP and CAD than group B (T2DM combined with insomnia group) and group C (insomnia combined with T2DM group). However, the prevalence of HBP and CAD was not significantly different between groups B and C. After adjusting for potential confounding factors, insomnia was significantly associated with HBP and CAD in patients with T2DM. These findings suggest that insomnia in patients with T2DM was more important in predicting the risk of macrovascular diseases such as HBP and CAD.

Insomnia is a complex disorder beyond short or disturbed sleep with clinical and therapeutic implications (13) and is associated with several metabolic disorders such as insulin resistance, obesity, and hypertriglyceridemia (14). In patients with obstructive sleep apnea, sleep loss concomitant with the hyperactivation of the sympathetic nervous system and intermittent hypoxia results in oxidative stress, inflammation, adipokine changes, and insulin resistance, increasing the risk of T2DM (15). This study showed that 36.4% of patients with T2DM had insomnia, consistent with previous research, indicating insomnia in 20–40% of patients with T2DM (16). Bani-Issa et al. (17) recruited 268 participants with T2DM from community healthcare settings in the UAE by using cluster sampling and reported that 34% of participants were poor sleepers and 55% of participants had “poor health-related quality of life.” A cross-sectional study on 504 Japanese patients with T2DM revealed that in individuals with T2DM, the prevalence of insomnia was high and some personality factors were involved (18). An interesting study showed that sleep deprivation in mice increased serum insulin, resistin concentrations, and weight (19). In another study, mice exposed to an 8-week protocol of sleep fragmentation exhibited increased calorie intake, with consequent obesogenic behavior and significant weight gain, implicating leptin resistance as an important factor (20). Furthermore, sleep fragmentation has been reported to result in insulin resistance through the activation of inflammatory pathways and oxidative stress (21).

The present results suggest that compared with group A (T2DM-alone group), groups B (T2DM combined with insomnia group), and C (insomnia combined with T2DM group) had a higher percentage of females (39.1 vs. 77.4%, *p* < 0.05; 39.1 vs. 55.2%, *p* < 0.05). This difference of gender still existed when groups B and C were compared (77.4 vs. 55.2%, *p* < 0.05). Group C had the highest level of HbA1c (*p* < 0.05 vs. group A), consistent with most previous studies (13, 16, 22, 23). In addition, the present findings suggest that group B had the highest level of BUN (*p* < 0.05 vs. group A) and Scr (*p* < 0.05 vs. groups A and C). It consistent with an earlier study by Yigit et al. indicating that elevated Scr levels were significantly related to excessive daytime sleepiness (24).

Previous studies have not determined the temporal sequence of insomnia and T2DM. Our findings suggest that 17.5% of patients with T2DM and insomnia had a longer duration of T2DM than insomnia. Furthermore, 18.9% of patients with T2DM and insomnia had a longer duration of insomnia than T2DM. The characteristics of the different temporal sequences of insomnia and T2DM have not been previously investigated. The present findings suggest that patients with T2DM combined with insomnia group generally had T2DM for > 15 years. On the other hand, the patients with insomnia combined with T2DM group generally had insomnia for > 10 years. Insomnia shares many risk factors with cardiovascular diseases (15, 25). These differences in the duration of insomnia and T2DM might have different effects on outcomes. Given the close relationship between insomnia and T2DM, we also investigated the relationship of insomnia with diabetic HBP and CAD in patients with T2DM.

This study indicated that patients in group A had a lower prevalence of HBP than those in groups B and C. The relationship between insomnia and HBP has been reported by previous studies; however, a causal relationship has not been established (26, 27). This study pointed to insomnia as an independent risk factor for HBP in T2DM population after adjusting for confounding variables. Several reports have supported this finding. A longitudinal study by Vgontzas et al. (28) indicated that individuals sleeping <5 h have the highest risk of HBP compared with those with normal hours of sleeping and those with > 6 h of sleeping (OR = 5.1, 95% CI: 2.2–11.8) followed by those sleeping 5–6 h (OR = 3.5, 95% CI: 1.6–7.9). The authors concluded that the risk for HBP was significantly higher in insomnia with a short sleep duration. Another multivariate longitudinal analysis suggested that insomnia severity in middle-aged adults was related to an increased risk of HBP with insomnia probably mediating the relationship between depression and hypertension (29).

This study suggests that patients with group A had a lower prevalence of CAD than the patients in groups B and C. Moreover, insomnia was an independent risk factor for CAD. In a large prospective cohort study by using the VACS by Polanka et al. (30), highly bothersome insomnia symptoms were significantly associated with CAD in HIV-infected veterans after adjustment for demographics, CAD risk factors, additional potential confounders, and HIV-specific factors. Grandner et al. (31) examined sleep disturbance as a predictor for the risk of cardiovascular and metabolic disorders in 138,201 individuals. They found that sleep disturbance was significantly associated with obesity, diabetes, CAD, and previous myocardial infarction and stroke. However, when physical health was included, the associations with diabetes and stroke were rendered non-significant.

The potential mechanisms of these connections have not been elucidated, but the autonomic nervous system (ANS) and endothelial dysfunction are suspicious targets. Previous sleep deprivation experiments have identified that blood pressure and sympathetic nervous system activity enhanced significantly after sleep restriction (32). Several researchers have also found elevated heart rates, increased metabolic rates, increased cortisol and norepinephrine concentrations, and elevated body temperatures in patients with chronic insomnia (33). Almost all the data indicate increased in sympathetic activity after partial or total acute sleep deprivation (34) or sleep fragmentation (35). Furthermore, a decrease in endothelium-dependent and endothelium-independent vascular reactivity was observed after acute total sleep deprivation, associated with increased levels of intercellular adhesion molecule 1, a marker of endothelial activation, and interleukin-6 (IL-6), which has been shown to inhibit endothelium-dependent nitric oxide-mediated relaxation (34). Other changes in patients with insomnia, such as oxidative stress, endothelial dysfunction, and prothrombotic state, are the same as those affected by sleep deprivation and might be the other potential mechanisms of these associations (36).

Most of the biological functions of the body change during sleep compared to the awake state such as heart rate (HR), arterial blood pressure (ABP), temperature, hormonal secretion, and immune function. Cardiovascular regulation is profoundly modified during sleep and the interconnection between the cardiovascular system and sleep processes must be considered as a bidirectional link. Cardiovascular diseases are associated with alterations of physiological sleep and vice versa sleep disorders can importantly alter the cardiovascular system, leading to an increased cardiovascular risk (37, 38). The above studies remind us that insulin resistance could be the potential connection between insomnia and cardiovascular disease. As a result, estimation for insomnia could identify individuals at high risk for HBP and CAD in patients with T2DM. However, the authors of the above studies did not determine the temporal sequence of insomnia and T2DM. Therefore, these discrepancies might be due in part to the temporal sequence of insomnia and T2DM. We found that group A had a lower prevalence of significant HBP than groups B and C, but there was no significant difference about the prevalence of HBP and CAD between group B and group C. Larger sample size studies are needed to validate the present findings.

This study demonstrated a prediction of cardiovascular diseases by insomnia in a T2DM population, revealing a causal relationship between them. However, some limitations need to be noted. The retrospective design (which might also be considered cross-sectional) of this study was the primary limitation. However, it did not limit our ability to comment on causality due to restrictions in the order of different diseases. Secondly, we did not carry out polysomnography to measure sleep; therefore, we could not accurately determine sleep disorders such as obstructive sleep apnea, which seems very common in patients with T2DM (39). Thirdly, the sample sizes in this study are relatively small, which may lead to a certain deviation in the results of this study. Finally, we did not collect data on (timing of) food intake, the timing of exercise, treatment adherence, and self-care, which might all be important, yet difficult to measure, potential confounding or explanatory variables. More prospective multicenter studies with larger sample sizes are required in a controlled setting.

In conclusion, this study indicates that insomnia is associated with an increased risk of HBP and CAD in patients with T2DM. This study might provide a new view of insomnia and new evidence on the different temporal sequence of insomnia and T2DM with diabetic cardiovascular complications. These associations warrant further investigations on whether sleep intervention could reduce these cardiovascular complications in patients with T2DM.

## Data Availability Statement

The raw data supporting the conclusions of this article will be made available by the authors, without undue reservation.

## Ethics Statement

The studies involving human participants were reviewed and approved by the Ethics Committee of Tianjin Medical University Chu Hsien-I Memorial Hospital. The patients/participants provided their written informed consent to participate in this study. Written informed consent was obtained from the individual(s) for the publication of any potentially identifiable images or data included in this article.

## Author Contributions

YH and CP wrote and designed the study. ZY contributed in the preparing of the manuscript. All authors reviewed the manuscript in detail, contributed to the article and approved the submitted version, and agree to be accountable for all the aspects of the work in ensuring that questions related to the accuracy and integrity of any part of the work are appropriately investigated and resolved.

## Funding

This study was supported by the Jiangsu Jiankang Vocational College Scientific Research Project (JKC201943).

## Conflict of Interest

The authors declare that the research was conducted in the absence of any commercial or financial relationships that could be construed as a potential conflict of interest.

## Publisher's Note

All claims expressed in this article are solely those of the authors and do not necessarily represent those of their affiliated organizations, or those of the publisher, the editors and the reviewers. Any product that may be evaluated in this article, or claim that may be made by its manufacturer, is not guaranteed or endorsed by the publisher.
